# Case of Strangulated Inguinal Hernia, in Which Three and a Half Inches of Mortified Intestine Were Removed by Excision

**Published:** 1863-01

**Authors:** H. B. Moore

**Affiliations:** Coldwater, Michigan


### CHICAGO MEDICAL JOURNAL.

VOL. VI.] JANUARY, 1863. [No. 1.

### ORIGINAL COMMUNICATIONS.

### CASE OF STRANGULATED INGUINAL HERNIA IN WHICH THREE AND A HALF INCHES OF MORTIFIED INTESTINE WERE REMOVED BY EXCISION.

By Dr. H. B. MOORE, of Coldwater, Michigan.

I was called on the 29th August last, to visit Mrs. Sophia Sperbeck, aged about 56, residing about four miles from town. Found her laboring under a Hernia, which for twenty-four hours past she and an assistant had assiduously endeavored to reduce. After an ineffectual attempt by taxis, to replace the tumor, she was put under a full opium treatment.

30th—Have failed to procure a decided narcotic effect from the treatment. Some disposition to vomit; pulse 80, without any febrile excitement, or much local soreness or pain; tumor hard, indicating omentum, though somewhat movable. Continued the treatment, two grains opium every two hours, until full effect should be obtained.

Next morning—31st—Dr. S. S. Cutter visited her in consultation. The patient has rested and feels but little uneasiness, indeed, no urgent symptoms except occasional vomiting. Failing to reduce the tumor by taxis, an operation was proposed, to which she readily assented. Upon carefully exposing and opening the sac, a portion of intestine, four inches over the external surface presented, the coats of which were soft emphysematous, black and fœtid, partially held in situ by adhesions, and containing a hard, impacted mass. Some hesitation now with regard to the best mode of procedure. To reduce the bowel in this condition would be certain death. To institute an artificial discharge would be, if successful, but little preferable. To remove it by excision was agreed on, which was accomplished by raising the intestine and dividing and removing the mortified portion entire down to the mesentery. The opposing ends were now brought together, and secured by four stitches, or interrupted sutures. The intestine was now returned within the abdomen. The external incision brought together and secured, and the patient removed from the lounge comfortably in bed: opiates and an emulsion of turpentine with spare diet constituted the principal subsequent treatment. Untoward symptoms, as vomiting, subsided. On the fifth day after the operation some fecal evacuation occurred, and again, three days thereafter, freely. Soon the bowels resumed their natural action, and now, Oct. 20, she considers herself well, with little disposition to hernial protrusion.

During the operation the patient was unconscious, from the use of chloroform, judiciously administered by Dr. Cutter, who also assisted in the operation.

Upon examining the portion of bowel removed, it was found to contain, besides some fecal matter, three dessert spoonsfull of blackberry seeds, four plum stones, and several fragments of chicken bone from one-fourth to five-eighths of an inch in length. The patient says she was accustomed to swallow her food imperfectly masticated, on account of defective and decayed teeth.

This may be regarded an unusual case, having but few precedents. It demonstrates the practicability of uniting inflamed membranous surfaces thickened by infiltration, between their coats, of abundant plastic lymph.

Although this method of practice may not be sustained by Gross, Miller and Druitt, yet it may be found fully supported by Dorsey and others of the older writers.

Dorsey says, vol. 2, page 56, "When the whole cylinder of the intestine is mortified, the mortified portion should be cut out, and the ends brought in contact and secured by means of four ligatures or sutures, one securing the mesentery and the three others at equal distances round the intestine. In every case, where the intestinal canal is partially destroyed by mortification, it is best to cut out the whole cylinder of the mortified part and not attempt to treat it only as a longitudinal wound, by removing only the portion actually gangrenous. There is a curious difference in the facility with which the longitudinal and transverse wounds of the intestines unite, the transverse heal readily while the longitudinal have a contrary tendency."

In Sam'l Cooper, part 2, page 295, says, "When the whole cylinder of the intestine is mortified the dead part is to be cut away, and the ends of the living are to be brought together and kept so by means of four stitches of fine thread or silk, then a thread is to be introduced through the mesentery. This is the only kind of suture that ought ever to be practiced on mortified intestine."

Charles Bells' Operative Surgery, vol. 1, page 198, recognizes the same practice, and says, "when a portion of intestine is mortified or gangrened, the discharge of the upper part of the canal should be allowed before an attempt is made to unite it by ligature."

In Lawrence, on Ruptures, page 242, etc., it is said, "if the divided bowel be approximated by sutures penetrating all the coats and cut off, a deposition of coagulating lymph unites its edges, and the sutures when loosened fall into the canal or are absorbed."

Many more treatises on Hernia might be quoted favoring the practice adopted in the foregoing case. I regard the opium treatment in this case, as having an important influence, counteracting the tendency to peritoneal inflammation.

